# Transurethral Catheterization in Early Training: The Impact of Peer-Led Mentorship

**DOI:** 10.1155/2021/8498835

**Published:** 2021-10-12

**Authors:** Mohamed Mubarak, Qasim Isa, Mahmood Alsaeed, Mohamed Alalawi

**Affiliations:** ^1^Department of Surgery, Salmaniya Medical Complex, Manama, Bahrain; ^2^Department of Medicine, Salmaniya Medical Complex, Manama, Bahrain

## Abstract

**Introduction:**

Transurethral catheterization (TUC) is a common hospital procedure. According to the literature, junior doctors contribute to the majority of TUC-related injuries. Our aim is to evaluate the immediate and long-term impact of a short procedure-centric TUC workshop on junior doctor's confidence, procedural knowledge, and ability to identify potential complications of catheterization.

**Materials and Methods:**

Intern doctors were invited to attend a one-hour workshop on TUC. A questionnaire was completed before and after the workshop. Three months later, the questionnaire was readministered to assess the workshop's long-term impact. The questionnaire consisted of three domains. A: experience, training, and confidence levels (using 5-point Likert scales), B: procedural knowledge (the highest possible score was 10 points), and C: identification of TUC-related complications (the highest possible score was 3 points).

**Results:**

81 interns participated and reported a confidence level of 3.03 ± 1.05 in performing a straightforward TUC. Preworkshop domain B and domain C were 3.92 ± 1.63 and 1.75 ± 0.69 points, respectively. After the workshop, reported confidence levels improved to 3.71 + 1.02 (*p* < 0.05). Likewise, the scores in domains B and C increased significantly to 8.85 ± 1.40 (*p* < 0.005) and 2.65 ± 0.6 (*p* < 0.005), respectively. Three months later, the same parameters were evaluated, and confidence levels were higher than those of the preworkshop levels at 3.83 ± 0.77 (*p* < 0.05). The average domain B score was 7.85 ± 1.88 (*p* < 0.005), and domain C score was 2.69 ± 0.53 (*p* < 0.005). All scores reported after three months were significantly better than the preworkshop levels (*p* < 0.005), but there were no statistically significant differences when compared to the immediate postworkshop scores (*p* > 0.05).

**Conclusion:**

Short peer-led TUC workshops positively impact intern doctors' confidence levels, procedural knowledge, and identifying complications.

## 1. Introduction

Transurethral catheterization (TUC) is a common hospital procedure, with up to 25% of all hospital patients being catheterized at one point of their admission [1]. Many studies indicate that, around 20% of in-patients and 7% of community-care patients have a urinary catheter [2]. Deficiencies in understanding the indications, procedure, and potential risks may result in injuries with significant short- and long-term complications [3, 4]. A yearlong study in an Irish tertiary-care teaching hospital revealed that 6% of all urology referrals were secondary to injuries from catheterizations performed by junior doctors [5]. Although considered a core skill, multiple studies have described that knowledge, confidence, and catheterization exposure were relatively substandard amongst junior doctors. Grimes et al. report that 76.1% of foundation doctors do not feel confident performing the procedure, with the majority not performing more than five catheterizations at the time of the study [6]. Forsythe et al. present similar results where around 55% of FY 1 and 2 doctors did not perform more than one male catheterization, with a higher proportion not performing a female catheterization [7]. Multiple other studies in France, Ireland, Nigeria, the Philippines, and the United Kingdom report similar outcomes [4–10].

In line with most internship programs globally and the foundation years program in the United Kingdom, it is expected for intern doctors to attain competency and perform TUC independently. It is also part of their internship logbook, which must be completed satisfactorily to complete the program [11, 12]. However, findings from the literature reflect the need for more procedural training and exposure. Educational theorists have highlighted that learning practical skills requires some form of mentorship in order to achieve competency. Miller et al. explain competency using a pyramid starting with learners knowing about a procedure and peaking with them performing it independently [13]. This can be facilitated for interns via judicious support through mentorship and structured training. One of the widely researched frameworks is Peyton's four-step approach (demonstrate, deconstruct, formulate, and perform). Peyton's approach to teaching procedures has been cited as superior to standard instruction in teaching technical skills [14]. Having understood the magnitude of the issue associated with performing catheterization at a junior level, we think implementing Peyton's approach in an informal peer-to-peer teaching setting could be an efficient and useful educational adjunct. This study evaluates the value of peer-led workshops in improving intern doctors' confidence, procedural knowledge, and awareness of procedure-related complications.

## 2. Materials and Methods

Intern doctors enrolled in the Ministry of Health's Internship Program in the Kingdom of Bahrain were invited to a one-hour workshop on the basics of catheterization for junior doctors. The contents and learning objectives of the workshop were discussed with urology consultants, and it was delivered by peers who have recently completed their internship program (junior residents/trainees) under the supervision of senior urology residents. It consisted of a lecture, a step-by-step video demonstration of the procedure, and a live simulated demonstration on a manikin. The lecture covered the following topics:

- Basic lower urinary tract anatomy
- Equipment used in catheterization
- Different types of catheters
- Indications and contraindications of catheterization
- Potential complications that could occur during or after the procedure

The interns were invited to complete a self-administered questionnaire before and after the workshop. Furthermore, another questionnaire was completed three months after the workshop to assess long-term retention. The questionnaire was adopted and modified from similar models used in previous studies [6, 9]. Each questionnaire consisted of 20 questions distributed across three main domains:

  A: experience and confidence
  B: procedural knowledge
  C: identification of complications

Domain A was measured using a 5-point Likert scale for confidence parameters. Performance in domains B (procedural knowledge) and C (identification of complications) was measured by calculating the number of correct answers from a total score of 10 and 3, respectively. Data are expressed as mean ± standard deviation, and statistical significance was set at *p* < 0.05. Analysis was conducted using the Statistical Package for Social Sciences (SPSS) v23. Measures of central tendency (mean, median, and mode) were calculated, and other statistical tests such as the *t*-test, ANOVA, and chi-square tests were performed where applicable.

## 3. Results

**Figure 1:**
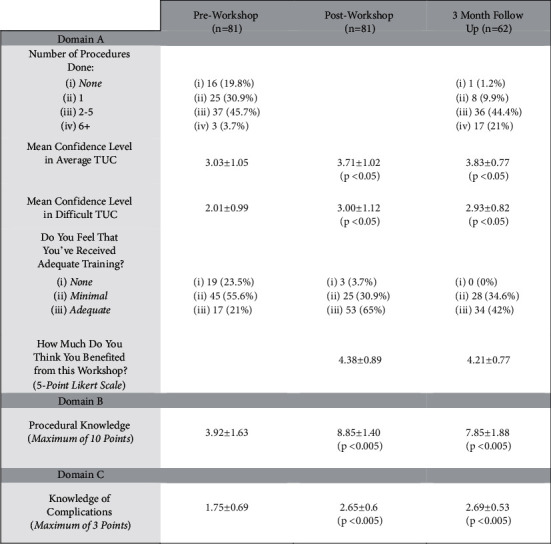
Summary of findings from the intern questionnaire (before and after the workshop and at 3-month follow-up).

**Figure 2:**
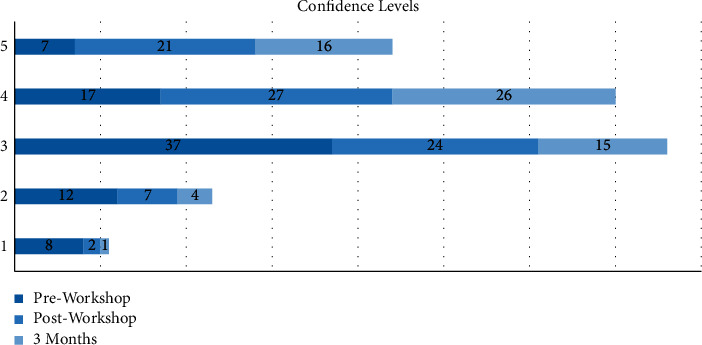
Reported confidence levels in performing an average difficulty TUC before and after the workshop and at three-month follow-up.

**Figure 3:**
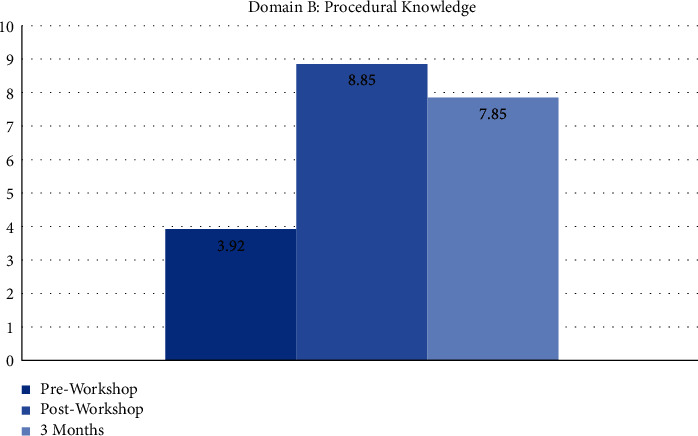
The average scores in domain B before and after the workshop and at three-month follow-up.

**Figure 4:**
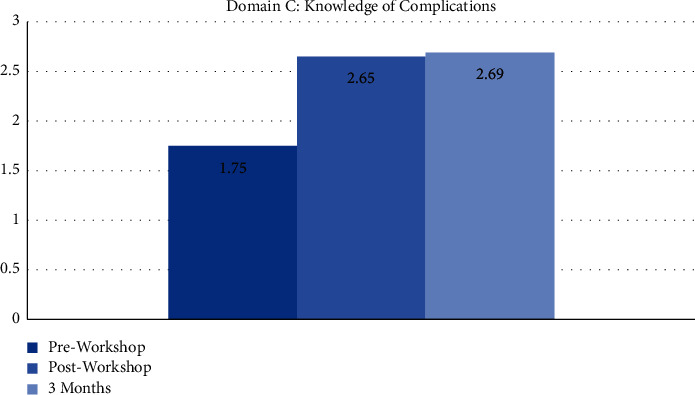
The average scores in domain C before and after the workshop and at three-month follow-up.

The workshop was attended by 81 interns who were at least six months into their internship program (Figure 1). Only 3 (3.7%) had performed more than five urethral catheterizations at the time, and only 17 (20.9%) felt they had adequate training. The majority of interns (74.4%) felt that a short workshop would improve their confidence levels. A preworkshop assessment of their confidence in performing an average difficulty and difficult catheterization using a 5-point Likert scale (1 = not confident and 5 = highly confident) showed an average score of 3.03 ± 1.05 and 2.01 ± 0.99, respectively (Figure 2). Procedural knowledge was scored out of a maximum of 10 points, and the average preworkshop score was 3.92 ± 1.63 points (range 0–9 points). Knowledge of identifying periprocedural complications was similarly graded out of a maximum of three points with an average score of 1.75 ± 0.69 points (Figures 1, 3, and 4). There was no significant correlation between the number of catheterizations performed in relation to procedural knowledge (*p* > 0.05) and the ability to identify complications (*p* > 0.05).

All 81 participating interns completed the postworkshop questionnaire (Figure 1). The mean confidence levels in performing an average difficulty catheterization rose to 3.71 ± 1.02 (*p* < 0.05), and confidence in performing a difficult catheterization increased to 3.00 ± 1.12 (*p* < 0.05) (Figure 2). There was a drastic change in both domains B and C of the questionnaire, with procedural knowledge scores increasing to 8.85 ± 1.40 (range 2–10 points) (*p* < 0.005) and recognition of complications scores increasing to 2.65 ± 0.6 (*p* < 0.005) (Figures 3 and 4). The majority of interns (90.1%) agreed the workshop as beneficial in improving their understanding of the procedure and its technicalities.

Three months after the workshop, we invited the participants to complete another questionnaire covering the same domains (Figure 1). Out of the original 81 participants, 62 responded (76.5%). The average number of procedures performed increased from 1.35 ± 0.89 to 2.16 ± 0.77 (*p* < 0.05), with around 21% having performed more than five catheterizations (Figure 1). On assessing their confidence with average and difficult catheterizations, a mean score of 3.83 ± 0.77 and 2.93 ± 0.82 points were recorded, thus showing a sustained improvement compared to preworkshop levels (*p* < 0.005) (Figure 2). In contrast, there were no statistically significant differences in both parameters compared to what was recorded immediately following the workshop (*p* > 0.05). Compared to the immediate postworkshop questionnaire, the average procedural knowledge dropped one point to around 7.85 ± 1.88 (*p* > 0.05) (Figure 3). Nevertheless, it was still much higher than the preworkshop scores (*p* < 0.005). In domain C, the average score was 2.69 ± 0.53 points, thus showing a maintained improvement from preworkshop levels (*p* < 0.005), but similar to the domains A and B, there was no significant difference compared to the immediate postworkshop levels (*p* > 0.05) (Figure 4).

## 4. Discussion

Although freshly graduated doctors are expected to be proficient in performing TUC, studies in the literature have highlighted deficiencies. As a basic bedside procedure, multiple factors play a role in determining the success of a TUC, such as experience, knowledge, confidence, and competence. Before our workshop, we wanted to assess our cohort's experience in performing the procedure. We found that, around half (50.7%) had performed either one or no procedures. Only three (3.7%) had catheterized more than five times at the time of our workshop. Similar results were described in a British multicenter survey involving 149 foundation year one (FY1) doctors, whereas 55.7% of FY1 doctors had performed fewer than five catheterizations. Only 15.5% had performed more than 10 at the time of the survey [6]. Likewise, an earlier study covering the same group of doctors in the United Kingdom also reported similar findings, with 29% of FY1 doctors having catheterized once only and 18% having never passed a catheter at all [10]. The literature generally shows a variation in the experience of intern doctors with the procedure. A recent Irish study surveying a cohort with a similar postgraduate experience as ours reported a significantly higher number of catheterizations with an average of 16 ± 7.8 catheterizations per doctor [15]. Other studies from the Philippines and Nigeria report higher procedure numbers as well [8, 9].

Being based in a high-volume hospital with 1300 beds, it was quite surprising to have a low number of catheterizations per intern, given that there is more than plenty to go around. However, we found that the low exposure to the procedure was related to both a lack of procedural knowledge and confidence amongst junior doctors. Before our workshop, most doctors reported average to low confidence levels in performing the procedure independently. Also, an astounding 79.1% felt that they received none to minimal training in the procedure and 74.1% believed they needed further training to feel confident. The findings were quite alarming, given that our cohort was halfway through their internship year at the time of the questionnaire. Unsurprisingly, similar findings were described in multiple studies across the globe, with reports of confidence rates ranging between 6% and 25% in similarly experienced groups of doctors [5, 6, 9, 15]. Other studies described a high confidence level amongst junior doctors coupled with high procedural volume; however, there were gaps in procedural knowledge and identifying complications that required fine-tuning despite the high confidence [8, 9]. Nevertheless, in our experience, low confidence amongst interns was most likely associated with poor procedural knowledge and practical experience.

The potential causes of these outcomes have been debated in previous studies. The most commonly cited reason has been inadequate exposure to urology during undergraduate medical education [15, 16]. However, the culprits in our experience were the combination of a poor induction program and a nonexistent mentorship scheme for interns. Both are indispensable tools that can introduce the procedure to interns and then gradually aid in developing their knowledge, confidence, and consequent competence in performing it. Another potential cause could be the impact of the COVID-19 pandemic on bedside teaching and training programs. Although previous intern batches could have had a better experience in the context of TUC, the pandemic put our cohort at a disadvantage. The recurrent deployment of both junior and senior doctors to COVID-19 centers made consistent bedside teaching and supervision difficult. This was reflected on the interns' perception of their training in TUC and need for further training. To counter this deficiency, the idea of peer-led catheterization workshops was conceptualized. In these workshops, junior trainees would deliver a short session with clear objectives and learning outcomes relevant to an intern doctor's scope of practice. To ensure quality, senior urology residents were present during the workshop, and the workshop's contents and learning objectives were discussed and approved by our urology consultants. After the workshop, interns reported an increase in confidence levels, which only decremented slightly over three months. Furthermore, factual knowledge of the procedure and its complications improved significantly as well, and satisfactory retention was seen on the three-month assessment. Both parameters are indicators of the positive impact of short peer-led workshops. Structured teaching, regardless of the delivery methods, has been shown to boost knowledge and confidence parameters in junior doctors. A recent study in France involving 40 intern doctors showed a statistically significant improvement (*p* < 0.05) in confidence levels after group sessions that included a presentation, a video demonstration, and simulation with peer feedback [17]. Benefits associated with these improvements also contribute to improvements in the quality of care and patient safety. In Ireland, Sullivan et al. described a successful experience by implementing a compulsory training module for intern doctors, leading to increased satisfaction with training and higher confidence levels in performing the procedure. They also report a decrease in complications associated with catheterization from 6% to 4% [18]. Similar improvements in morbidity rates were reported by an American study that involved a cohort of nurses [19]. These findings from the literature are essential to emphasize on, as it has been reported that 73% of all catheter-related complications were related to procedures performed by intern doctors [5].

Devising structured teaching to train intern doctors in practical procedures requires significant resources. However, its positive outcomes on improving competence levels and its effect on patient safety instigate us to use the arsenal of teaching and instruction alternatives available. Peer-led workshops are an efficient and cost-effective method that can be used to deliver teaching and involve junior doctors in peer-to-peer mentorship. In our experience, the advantages of this method extend to promoting peer mentorship relationships and fostering a more casual teaching environment, which makes the transition for interns easier. Other modalities available include simulation workshops or courses. Although they are better known for teaching technically complex procedures such as laparoscopy or endoscopy, they are excellent tools for teaching bedside procedures such as TUC. Todsen et al. report that medical students demonstrate good transfer of catheterization skills from the lab to actual clinical practice. Accordingly, they recommend simulation to be a standard in all medical school curricula [20]. YouTube has also been implicated as a potential learning resource; however, a Swedish study reviewing the material available in 2014 concluded that the quality is variable and poor [21].

An important limitation of our study is the high variability in the clinical experience of our intern cohort. The workshops were carried out six months into the internship program, and not all interns had rotated in major hospital-based rotations such as medicine and surgery. Also, there were a few interns who spent a part of their major rotations in deployment. Unfortunately, we could not extend our questionnaire to include female TUC, as the procedure is performed by female nurses due to sociocultural conservations. Recall bias is another limitation as our data were based on questionnaires. Lastly, given that the confidence levels were based on self-assessments, there is a risk of bias in terms of over- or underscoring [22, 23]. To further validate the results, we encourage direct supervision by trainers or cross assessment by peers in order to identify and calibrate any discrepancies in scoring.

## 5. Conclusions

Short peer-led workshops on transurethral catheterization effectively improve intern doctors' confidence levels, procedural knowledge, and ability to identify complications. Given the challenges posed by the COVID-19 pandemic on traditional bedside teaching, we encourage implementing such workshops. They are easy to organize, require minimal resources, and can help build mentee-mentor relationships amongst junior doctors, all while providing a casual atmosphere that produces maintained improvements in the long run. Moreover, we highly encourage incorporating other methods such as simulation, dry labs, and boot camps into the intern training program as well. This will help further their confidence levels and competency in these early stages of their careers.

## Data Availability

The data used to support the findings of this study are included within the article and supplementary file.

## Supplementary Materials

Supplementary Materials Appendix 1: the questionnaire used in the assessments.
