# Supplementary material for: Transurethral Catheterization in Early Training: The Impact of Peer-Led Mentorship
Source: Surg Res Pract. 2021 Oct 12;2021:8498835. doi: 10.1155/2021/8498835 (PMC8526240; doi:10.1155/2021/8498835)
Supplement: Supplementary Materials — Appendix 1: the questionnaire used in the assessments. [file 8498835.f1.pdf]

Transurethral Catheterisation for Junior Doctors

Please enter your email address correctly & select your gender.

If you have any questions regarding any of the questions in this survey feel free to ask.

* Required

1. E-Mail Address *

2. Gender *

Mark only one oval.

- Male
- Female

Urethral Catheterization for Junior Doctors

Pre-Workshop Assessment: Previous Experience

Please answer all of the questions below to the best of your knowledge.

3. How many urethral catheterizations have you performed? *

Mark only one oval.

- 0
- 1
- 2-5
- 6-10
- >10

4. On a scale of 5, how confident do you feel about performing a urethral catheterization of average difficulty (1 = not confident, 5 = high confident) *

Mark only one oval.

1	2	3	4	5

5. On a scale of 5, how confident do you feel about performing a difficult urethral catheterization. In this context, difficult means that other medical staff attempted to but failed to catheterize before calling you. (1 = not confident, 5 = high confident) *

Mark only one oval.

1	2	3	4	5
◯	◯	◯	◯	◯

6. Do you feel that you have received adequate training on urethral catheterization? *

Mark only one oval.

- ◯ Adequate
- ◯ Minimal
- ◯ None

7. On a scale of 5, how do you think that a short workshop on catheterization skills would improve your confidence levels? (1 = will not improve, 5= highly improve) *

Mark only one oval.

1	2	3	4	5
◯	◯	◯	◯	◯

Urethral Catheterization for Junior Doctors

Pre-Workshop Assessment.

Please answer all of the questions below to the best of your knowledge. This is not a test.

8. Do you take history from the patient prior to urethral catheterization? *

Mark only one oval.

- ◯ Yes
- ◯ No
- ◯ Sometimes

9. Do you explain the procedure to the patient prior to urethral catheterization? *

Mark only one oval.

- ◯ Yes
- ◯ No
- ◯ Sometimes

10. What method of lubrication should be used when catheterizing a patient? *

Mark only one oval.

- ◯ Applying lubricant to the catheter

- Applying lubricant to the catheter
- Applying lubricant to penile meatus
- Injecting lubricant into the urethra
- No lubrication is usually required

11. At what angle, relative to the patient's body, should the penis be stretched when inserting the catheter? *

Mark only one oval.

- Parallel (180 degrees) to the body
- Angled (45 degrees) relative to the body
- Perpendicular (90 degrees) to the body
- No particular angle

12. How deep should the catheter be inserted before you inflate it balloon? *

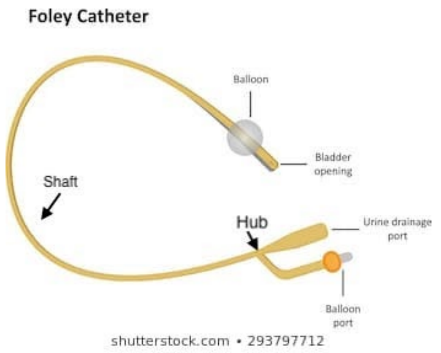

Mark only one oval.

- Once half of the catheter's length is in
- As soon as urine appears, regardless of catheter depth
- Till the hub (end of the catheter, where the balloon port is located)
- None of the above

13. Which of the following is the most suitable solution to inflate the catheter balloon with? *

Mark only one oval.

- Normal Saline
- Air
- Distilled Water
- Tap Water
- None of the above

14. What is the most suitable catheter size for an average female? *

Mark only one oval.

- 8-10 French
- 10-12 French
- 12-14 French
- 14-18 French
- 20-22 French

15. What is the most suitable catheter size for an average male? *

Mark only one oval.

- 8-10 French
- 10-12 French
- 12-14 French
- 14-18 French
- 20-22 French

16. 1 French = __ mm *

Mark only one oval.

- 0.1 mm
- 0.25 mm
- 0.33 mm
- 0.45 mm
- 0.5 mm

17. Which of the following is not an indication for inserting a three-way catheter? *

Mark only one oval.

- Post-Transurethal Surgery
- Urinary Tract Infection
- Hematuria
- Bladder Wash & Irrigation
- All of the above

18. You are catheterizing a 52 year old male with no comorbidities. You successfully insert the catheter and inflate the balloon as soon as urine drains. Immediately, blood starts draining from the catheter's tip. What is the most likely cause? *

Mark only one oval.

- Bladder perforation
- Normal insertion
- Urethral injury
- Balloon not inflated properly
- None of the above

19. You were called regarding an 80 year old male whom you inserted a catheter for 2 hours

ago. The staff inform you that he was passing urine when you kept the catheter for him, but now he is not passing any urine. What could be the cause? *

Mark only one oval.

- Catheter is in the urethra
- Bladder obstruction
- Bladder perforation
- Empty bladder
- None of the above

20. Before discharging a patient you are requested to remove the catheter. During the procedure, you try to deflate the balloon, however, it does not deflate and therefore you are unable to pull it out. What could be the cause? *

Mark only one oval.

- Kinked catheter
- Crystallization of balloon fluid
- Spasm of the external urethral sphincter
- Misplaced catheter in the urethra
- None of the above

This content is neither created nor endorsed by Google.

Google Forms
